# Cytological Analysis of Crossover Frequency and Distribution in Male Meiosis of Cardueline Finches (Fringillidae, Aves)

**DOI:** 10.3390/ani13233624

**Published:** 2023-11-23

**Authors:** Ekaterina Grishko, Lyubov Malinovskaya, Anastasia Slobodchikova, Artemiy Kotelnikov, Anna Torgasheva, Pavel Borodin

**Affiliations:** Department of Molecular Genetics, Cell Biology and Bioinformatics, Institute of Cytology and Genetics, 630090 Novosibirsk, Russia; grishko@bionet.nsc.ru (E.G.); malinovskaya@bionet.nsc.ru (L.M.); a.slobodchikova@alumni.nsu.ru (A.S.); a.kotelnikov@g.nsu.ru (A.K.); torgasheva@bionet.nsc.ru (A.T.)

**Keywords:** crossing over, interference, metapolycentromeres, meta-polycentromeres, centromere effect

## Abstract

**Simple Summary:**

Meiotic recombination, which involves the reshuffling of genes, plays an important role in generating biodiversity. However, studies on this process in bird chromosomes are scarce. Using antibodies targeting proteins involved in recombination, we examined the number of recombination events across the entire genome and their distribution along the largest chromosome in the germ cells of four closely related songbird species: *Common linnet*, *Eurasian bullfinch*, *Eurasian siskin*, and *European goldfinch*. We found significant variance in the frequency of recombination events among these species, as well as individual variability within each species. Across all four species, the distribution of recombination events on chromosome 1 was remarkably consistent, with more events occurring toward the chromosome ends and fewer near the centromere. The proximity of these events to the centromere depended on how many events took place on that chromosome, with more events being associated with closer locations. Interestingly, the size and type of the centromere did not influence this pattern. We propose that the scarcity of recombination events near the centromere may result from their sequential occurrence along the chromosome, starting from the chromosome ends, rather than from any specific influence from the centromere itself.

**Abstract:**

Meiotic recombination is an important source of genetic diversity. Using immunolocalization of several meiotic proteins at the spreads of male pachytene cells, we estimated the number of recombination nodules per cell and their distribution along the macrochromosome 1 of the *Common linnet*, *Eurasian bullfinch*, *Eurasian siskin*, and *European goldfinch*. The macrochromosomes of the two former species have metapolycentromeres, composed of several centromeric domains. We detected significant interspecies differences in the mean numbers of recombination nodules per genome: 52.9 ± 2.8 in the *linnet*, 49.5 ± 3.5 in the *bullfinch*, 61.5 ± 6.3 in the *siskin* and 52.2 ± 2.7 in the *goldfinch*. Recombination patterns on macrochromosome 1 were similar across species, with more nodules localized near chromosome ends and fewer around centromeres. The distance from the proximal nodule to the centromere depended on the nodule count per chromosome arm, with more events leading to a closer location. However, species with different centromere types showed no difference in this regard. We propose that the deficiency of recombination sites near centromeres could be due to the sequential occurrence of crossovers starting from the chromosome ends and may not be attributed to any suppressive effect of the centromere itself.

## 1. Introduction

Meiotic recombination, the process of reciprocal genetic exchange between homologous chromosomes during meiosis, influences adaptation and speciation [1,2,3].

Recombination involves the programmed generation of multiple double-strand breaks of DNA (DSBs) at the beginning of meiotic prophase (the leptotene stage) and their repair in crossover or non-crossover pathways during the zygotene and pachytene stages. Thus, the recombination rate depends on the number of DSBs generated and those repaired via the crossover pathway [4,5,6].

The distribution of meiotic recombination events along chromosomes is not random. At the genomic level, it is controlled by the localization of recombination hotspots where the frequency of DSB might be several orders of magnitude higher than in the other regions [7,8,9,10]. The activity of hotspots is modulated by chromatin properties. Recombination is generally reduced in more compact genomic regions (heterochromatin) compared to less compact regions (euchromatin) in almost all studied organisms [11].

The process by which the repair pathway for DSBs is chosen remains unknown. However, this process is regulated at the chromosome level, as the global pattern of recombination event distribution on chromosomes shares common features (a pronounced telomeric peak, reduced (or absent) recombination in centromeric regions) among the best-studied organisms [3,12,13]. It has been suggested that the pattern of crossing-over distribution along the chromosomes is determined by three factors: assurance, interference, and the centromere effect [13]. Assurance presumes a requirement of at least one crossover per bivalent (obligate chiasma) necessary for orderly chromosome segregation at the first meiotic division [14]. Interference decreases the probability of recombination events close to other events [15]. The centromere effect indicates a suppression of recombination near the centromere [16].

The factors controlling recombination frequency and distribution have been extensively studied in mammals [17,18,19,20]. Recombination properties of avian chromosomes attracted less attention [21,22,23] though several peculiarities of bird genomes make them promising for such studies. Their karyotypes are bimodal, comprising several macrochromosomes and dozens of microchromosomes. They are extremely conservative in terms of chromosome number and content. Strong chromosomal synteny between different bird species and even between birds and reptiles indicates that interchromosomal rearrangements, such as translocations, were rarely fixed during their evolution, while intrachromosomal rearrangements, such as inversions and centromere shifts, were abundant [24,25,26,27,28]. Recently, metapolycentromeres have been described in several bird species [29]. These structures are composed of several consecutive domains of centromeric heterochromatin and function as a single centromere [30]. This combination of karyotype stability and variation of genetic content between orthologous chromosomes might help to reveal and differentiate large and fine-scale factors controlling recombination rate and distribution.

**Figure 1:**
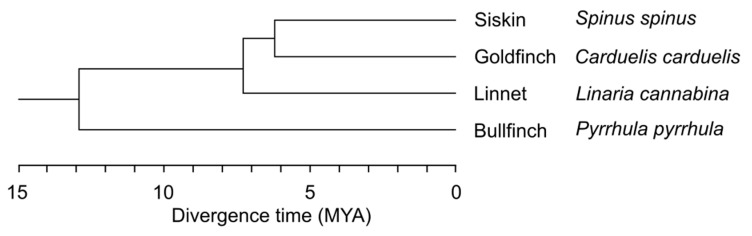
Cladogram of cardueline finches (according to [31]).

In this paper, we examine the recombination frequency and its distribution along the chromosomes of four closely related species of cardueline finches (Fringillidae, Passeriformes) (Figure 1). They have the same chromosome number (2n = 82), comprising seven pairs of macrochromosomes and 34 pairs of microchromosomes [29]. All chromosomes of the *Eurasian siskin* (*Spinus spinus)* and *European goldfinch* (*Carduelis carduelis*) have regional centromeres [29] while several macrochromosomes of the *Common linnet* (*Linaria cannabina*) and *Eurasian bullfinch* (*Pyrrhula pyrrhula*) have metapolycentromeres (E. Grishko, unpublished observations). The effect of metapolycentromeres on recombination patterning has not been studied.

To visualize recombination events along the meiotic chromosomes, we used immunolocalization of MLH1, the mismatch repair protein marking mature recombination nodules, SYCP3, the main protein of lateral elements of synaptonemal complexes (SC) and human centromere proteins, marking bird centromeres. This approach has been successfully applied to recombination studies of birds [21] and mammals [19].

We addressed the following questions:

Do the bird species, which are recently diverged [31] and have similar karyotypes [30], differ in recombination frequency and distribution? The magnitude of the differences might indicate the speed of the evolution of the traits [32]. The direction of the changes could help to estimate whether they are adaptive or not [2,33].

What is the role of crossover interference and centromere effects in controlling the distribution of recombination along the chromosomes? A substantial contribution of these factors has been convincingly demonstrated in the cytological analysis of mammalian meiosis [19], but remains to be analyzed in birds [21].

Does the centromere type affect recombination frequency and distribution? Metapolycentromeres are cytogenetic phenomena, and their effects on chromosome function are largely unknown [30].

## 2. Materials and Methods

### 2.1. Specimens

Adult males were sampled during the breeding season (April–May). They were provided by the Animal Rehabilitation Centre (Novosibirsk) after fatal accident traumas. The handling and euthanasia of the birds were conducted in compliance with approved national guidelines for laboratory animal care and use. Euthanization was performed by administering an isoflurane overdose. The study adheres to the ARRIVE guidelines (https://arriveguidelines.org accessed on 10 October 2023) and was approved by the Animal Care and Use Committee of the Institute of Cytology and Genetics SB RAS (protocol # 114).

### 2.2. Synaptonemal Complex Spreading and Immunostaining

We prepared SC spreads from the freshly isolated testes using the drying-down method described by Peters et al. [34] and immunostained them following the protocol outlined by Anderson et al. [35]. The slides were incubated overnight at 4 °C in a humid chamber with primary antibodies, including rabbit polyclonal anti-SYCP3 (1:500; Abcam, Cambridge, UK), mouse monoclonal anti-MLH1 (1:30; Abcam, Cambridge, UK), rabbit polyclonal anti-H3K9me3 (1:100; Abcam, Cambridge, UK), and human anticentromere (ACA) (1:70; Antibodies Inc., Davis, CA, USA). Secondary antibody incubations were performed at 37 °C for 1 h using Cy3-conjugated goat anti-rabbit (1:500; Jackson ImmunoResearch, West Grove, PA, USA), fluorescein isothiocyanate (FITC)-conjugated goat anti-mouse (1:30; Jackson ImmunoResearch, West Grove, PA, USA), fluorescein isothiocyanate (FITC)-conjugated donkey anti-rabbit (1:30; Jackson ImmunoResearch, West Grove, PA, USA) and aminomethylcoumarin (AMCA)-conjugated donkey anti-human (1:40; Jackson ImmunoResearch, West Grove, PA, USA). Non-specific antibody binding was blocked using a 10% PBT solution. Vectashield antifade mounting medium (Vector Laboratories, Burlingame, CA, USA) was applied to minimize fluorescence fading. An Axioplan 2 microscope (Carl Zeiss, Jena, Germany) equipped with a CCD camera (CV M300, JAI Corporation, Yokohama, Japan), CHROMA filter sets, and the ISIS4 image-processing package (MetaSystems GmbH, Altlußheim, Germany) was used for visualization.

### 2.3. Image Analysis

We used MicroMeasure 3.3 software [36] to measure the length of each SC arm in micrometers. We scored the number of MLH1 signals localized on SCs and their distances to centromere boundaries (distal ends of the centromere signals). The SC of the largest submetacentric macrochromosome 1 was unambiguously identified by its relative length and centromeric index. To map the MLH1 foci distribution along the SC, we plotted the relative number (proportion) of MLH1 foci within 1 μm intervals of the average SC length.

### 2.4. Statistical Analysis

Descriptive statistics were performed using the Python packages statsmodels [37] and SciPy [38]. The one-way ANOVA was employed to assess differences between the species and individuals in terms of the average SC length and number of MLH1 foci. A significance level of *p* < 0.01 was considered statistically significant. Mean ± standard deviation (S.D.) values are reported in the text and figures.

## 3. Results

### 3.1. Total Recombination Rate per Cell

**Figure 2:**
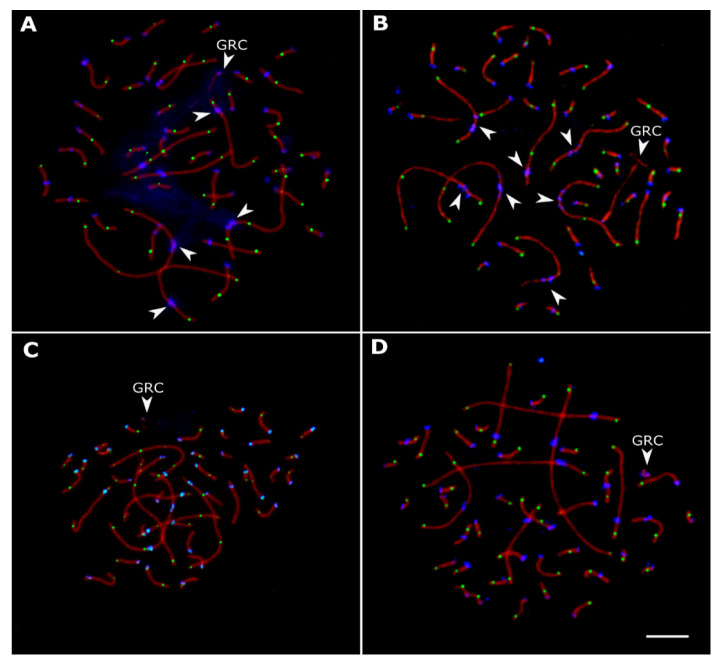
Pachytene spermatocytes of *linnet* (**A**), *bullfinch* (**B**), *siskin* (**C**), and *goldfinch* (**D**) after immunolocalization of SYCP3 (red), MLH1 (green) and centromere proteins (blue). Arrowheads indicate metapolycentromeres and GRC. Scale bar: 5 μm.

A total of 573 pachytene spermatocytes were analyzed. Figure 2 shows representative pachytene spermatocytes of *linnet* (A), *bullfinch* (B), *siskin* (C), and *goldfinch* (D). All pachytene cells contained 41 bivalents and a univalent representing the additional germline-restricted chromosome (GRC) characteristic of all examined passerine birds [39]. Recombination-related traits of the species examined are shown in Table 1. In all birds, the observed number of MLH1 foci was much higher than the minimum of 41 foci required for ensuring at least one chiasma per bivalent for proper meiotic segregation of homologous chromosomes. Most macrochromosomes contained at least one MLH1 focus at each arm.

The ANOVA revealed significant interspecies (F = 244.1, *p* < 0.01) and inter-individual (*p* < 0.01 for all four species) differences in the total recombination rate estimated as the mean number of MLH1 foci per cell. The pairwise comparison demonstrated significant differences in this trait between all species examined (Student’s *t*-test, *p* < 0.01). *Siskin* showed the highest value, and *bullfinch* showed the lowest. There was also significant interspecies (F = 122.7, *p* < 0.01) and inter-individual (*p* < 0.01 for all four species) variation in the total length of SC. Male *linnets* had the largest value, *goldfinch* had the lowest. The coefficients of correlation between the total number of MLH1 foci and total SC length were 0.57 in *linnet*, 0.45 in *bullfinch*, 0.64 in *siskin*, and 0.27 in *goldfinch* (*p* < 0.01).

**Figure 3:**
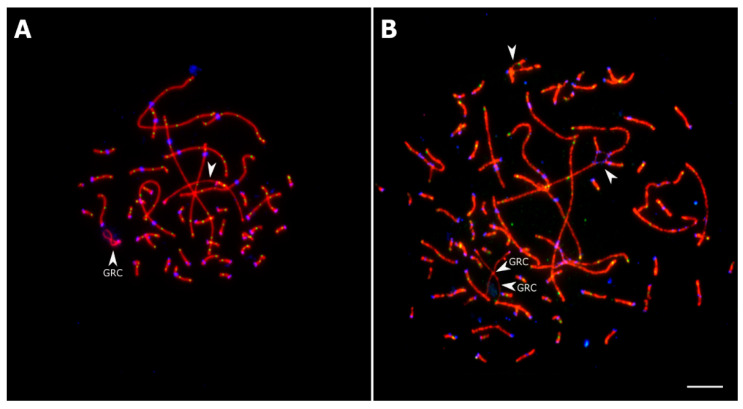
Exceptional pachytene cells of the *goldfinch* (**A**) and the *bullfinch* (**B**) after immunolocalization of SYCP3 (red), MLH1 (green), and centromere proteins (blue). Arrowheads indicate GRCs, a univalent of an additional chromosome (**A**), and multivalents (**B**). Scale bar: 5 μm.

In the *goldfinch* and *bullfinch*, we observed several exceptional cells, which were excluded from the analysis. Four pachytenes of one *goldfinch* specimen contained atypically long GRC and an unidentified univalent. The total MLH1 count in these cells was 1.5 times higher than the average for the *goldfinch* (Figure 3A). In one *bullfinch* specimen, we found several tetra- and octoploid cells with aberrant chromosome synapsis resulting in multivalents (Figure 3B).

### 3.2. Variations in Centromere Structure

Malinovskaya et al. [29] described metapolycentromeres in several chromosomes of the European pied flycatcher, gouldian finch, and domestic canary. We also observed metapolycentromeres in the *linnet* and *bullfinch* (Figure 2A,B). In the *linnet*, they were present in the three largest SCs and in one of the smaller macrochromosomes. The large SCs contained two or three centromeric domains, which covered about 5% of the SC length. In the *bullfinch*, the metapolycentromeres were present in all seven macro-SCs. They consisted of two or three domains and covered about 10% of the SC length.

**Figure 4:**
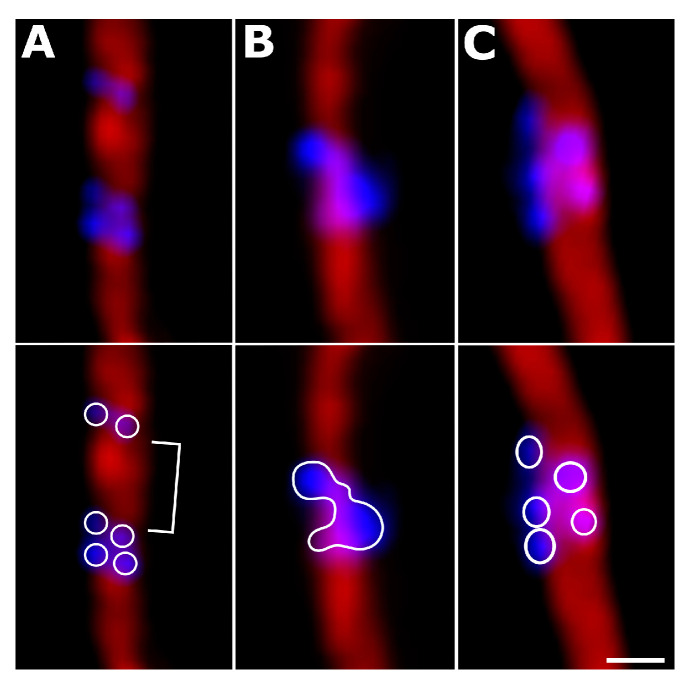
Variation of metapolycentromere structure in the *bullfinch* (**A**,**B**) and *linnet* (**C**). (**A**) gap between the centromeric protein-associated domains; (**B**) fusion of centromeric domains; (**C**) unequal number of domains at the homologous chromosomes. Scale bar: 0.5 µm.

The *linnet* and *bullfinch* metapolycentromere domains formed a paired bead-like pattern along the SC, typical for all metapolycentromeres described so far (Figure 4) [23,30,40,41]. Some metapolycentromeres demonstrated large gaps between the domains (Figure 4A). We also revealed a fusion of centromeric protein-associated domains (Figure 4B) and different numbers of domains on the two lateral elements of a SC. For instance, while one of the lateral elements contained three domains, the other one contained two domains (Figure 4C). This might indicate heterozygosity for the domain number.

**Figure 5:**
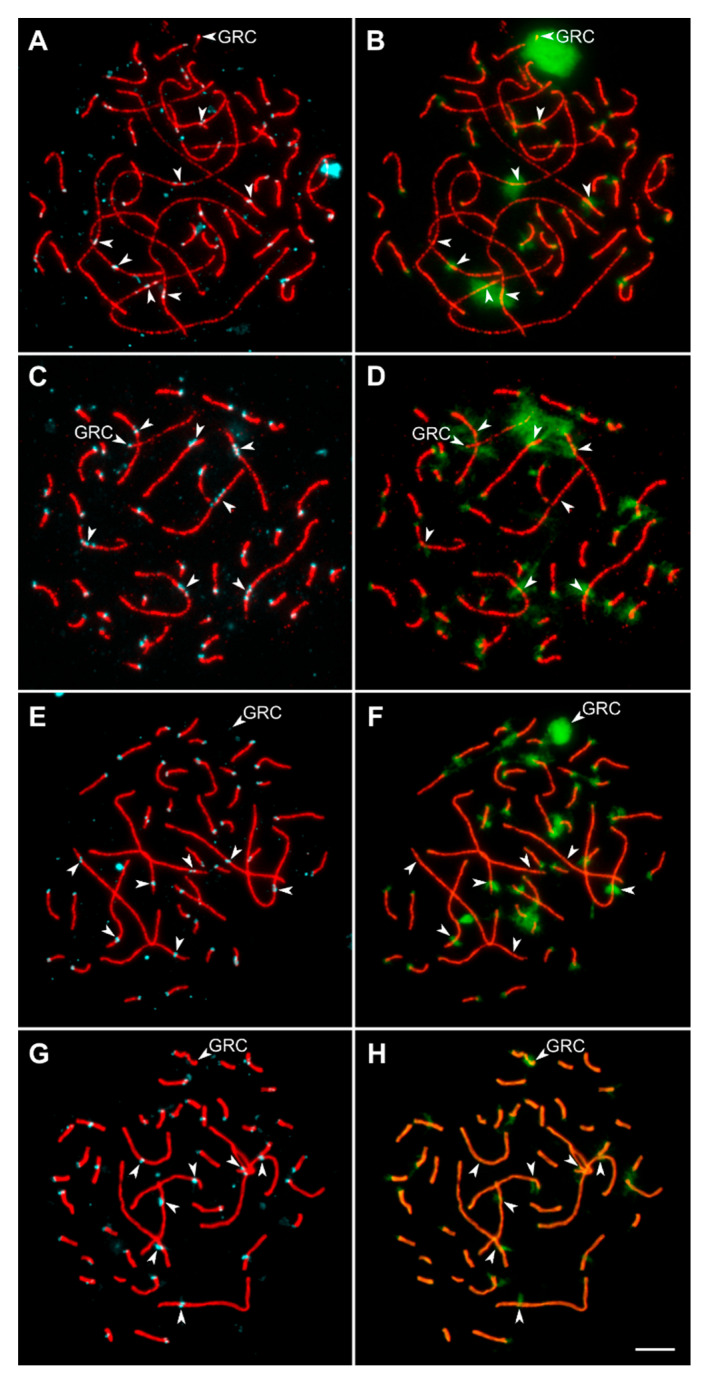
Pachytene spermatocytes of the *linnet* (**A**,**B**), *bullfinch* (**C**,**D**), *siskin* (**E**,**F**), and *goldfinch* (**G**,**H**) after immunolocalization of SYCP3 (red), centromere proteins (blue) (**A**,**C**,**E**,**G**), and H3K9me3 (green) (**B**,**D**,**F**,**H**). Arrowheads point to the seven largest macrochromosomes and GRC. Scale bar: 5 μm.

Antibodies against H3K9me3 labeled both the regional and metapolycentromeres of the *linnet*, *bullfinch*, and *goldfinch*, except for one of the macro-SCs (Figure 5). In the *siskin*, we detected H3K9me3 labeling at three macro-SCs (Figure 5E,F). In the *linnet*, the unlabeled SC was a metacentric macro-SC with a regional centromere (Figure 5A,B). In the *bullfinch*, it was a submetacentric macro-SC with metapolycentromere. We also detected H3K9me3 labeling at the ends of some macro-SCs in the *bullfinch* (Figure 5C,D). In the *goldfinch*, H3K9me3 labeling was absent on a metacentric macrochromosome (Figure 5G,H). In the *siskin*, among four macro-SCs with absent H3K9me3 labeling, one macro-SC was metacentric. Given that the Z chromosome is known to be middle-sized metacentric in the *siskin* and *goldfinch* [42] we assume that the Z chromosome is also metacentric in the *linnet* and submetacentric in the *bullfinch*. Thus, we suppose that, in all four species, the centromeres of the ZZ bivalent are H3K9me3-negative. In the *linnet*, *bullfinch*, *siskin*, and *goldfinch*, 31 out of 34, 33 out of 34, 34 out of 34, and 20 out of 34, respectively, micro-SC centromeres were H3K9me3-positive. In all four species, we detected H3K9me3 labeling on GRCs. In the *linnet*, *siskin*, and *goldfinch*, the GRC was heavily marked by a cloud of H3K9me3 antibodies. In the *bullfinch*, H3K9me3 labeling appeared only on a distal part of the GRC.

### 3.3. Recombination Distribution along SC1

To investigate how recombination features of the same chromosome vary across different species and whether the centromere type affects this variation, we estimated SC length, recombination frequency and distribution, and parameters of interference on the short and long arms of SC1. We chose this bivalent because we were able to identify it unambiguously by its relative length and centromeric index in all four species. We supposed that it was the SC of chromosome 1: the largest chromosome in the karyotypes. Among songbirds, it is conserved in such phylogenetically distant species as the domestic canary and the zebra finch [43].

Table 2 shows recombination-related traits for arms of the SC1 in the species examined. The highest number of MLH1 foci per SC1 was observed in the *siskin*, the species that showed the highest total MLH1 number.

Chiasma interference is an important factor affecting the number and distribution of the recombination events along the chromosomes; an occurrence of one recombination event suppresses the probability of another event in its vicinity [44]. In terms of MLH1 foci, which we used as the markers of recombination sites, the positions of the neighboring foci should be interdependent. The distances from the centromere to neighboring MLH1 foci within the same arm should then be positively correlated: the further one focus is from the centromere; the further the neighboring one should be located. MLH1 foci separated by the centromere should show a negative correlation: the closer a focus is to the centromere on the short arm, the further it is from the centromere on the long arm, and vice versa [45].

Table 3 shows the coefficients of correlation between the distances from the centromere to the neighboring MLH1 foci within the arm and across the centromere in the SC1. Most correlation coefficients within the arms are positive, rather high, and significant (*p* < 0.01). The low and insignificant coefficient in the *goldfinch* is due to the low number of multiple MLH1 foci at their arms (Table 2). In contrast, all correlation coefficients between the distances from the centromere to the foci separated by the centromere were close to zero and non-significant. This means that the chiasma interference in the examined bivalents was strong and significant within the arms and negligible across the centromere.

Given the absence of chiasma interference across the centromere, we estimated the degree of interference for each arm separately using the relative distances between two neighboring MLH1 foci (Table 2). These distances varied from one-third to two-thirds of the arm’s length. The longer arms accommodated more MLH1 foci, which were located close to each other.

**Figure 6:**
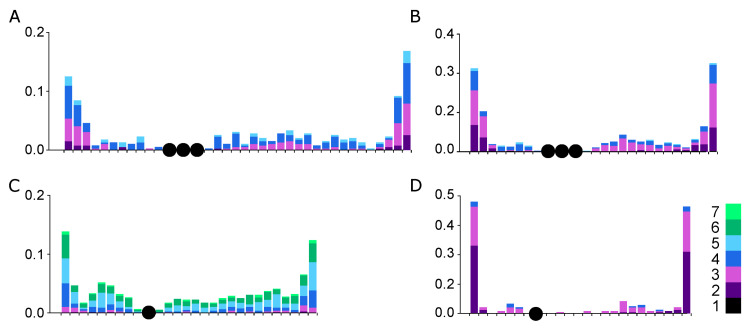
Distribution of MLH1 foci along the SC1 in the pachytene cells of the *linnet* (**A**), *bullfinch* (**B**), *siskin* (**C**), and *goldfinch* (**D**) The X-axis reflects the position of the foci in the SC relative to the centromere, indicated by a single circle (the regional centromere) or multiple circles (metapolycentromeres). Each interval represents 1 µm of the average SC length. The Y-axis reflects the proportion of MLH1 foci in each interval. The colors represent the proportion of bivalents with 1 to 7 MLH1 foci per chromosome.

Interference affected the distribution of MLH1 foci along the SC1 in all species examined, as shown in Figure 6. The recombination landscapes of the SC1 of the cardueline finches are rather similar to those described in other birds analyzed both cytologically [23,46] and genetically [47,48]. Clear peaks of MLH1 foci are located at the SC ends while the pericentromeric area of most SCs contains few or no foci.

**Figure 7:**
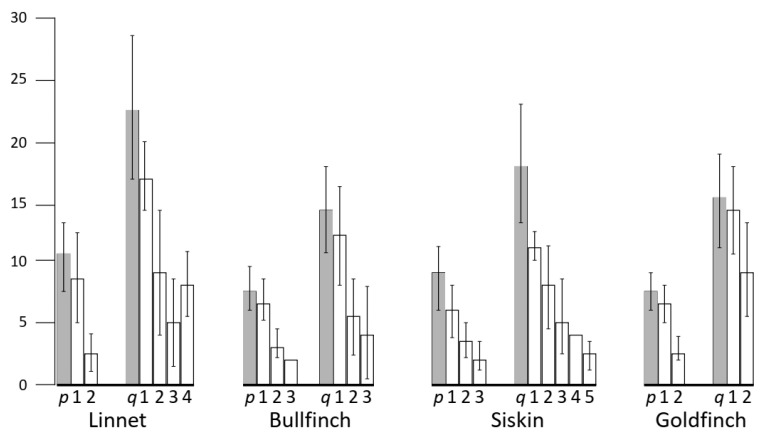
The distance from the proximal MLH1 foci to the centromere (or centromere boundary in the species with metapolycentromeres) on the SC1 arms with different numbers of MLH1 foci. The Y-axis shows the average (±S.D.) distances (µm) from the proximal MLH1 foci to the centromere (white columns) compared to the length (±S.D.) of the short (p) and long (q) arms of the SC1 (grey columns). The numbers below the gray columns indicate the number of MLH1 foci per arm.

To estimate the centromere effect, we assessed the distance from the proximal MLH1 foci to the centromere on each arm. Figure 7 shows that this distance decreases with the increase in the number of MLH1 foci per arm. Single foci are usually located in the peritelomeric regions close to the arm ends. The more foci occur on the arm, the closer to the centromere proximal foci are located. In many cells with multiple MLH1 foci, the proximal one was located on the boundary of the centromere region or very close to it. Species with regional and metapolycentromeres did not differ in the average distance to the centromere from the proximal MLH1 foci (Figure 7).

## 4. Discussion

The total recombination rates of the cardueline species examined here fall within the limits of variation for this trait among the bird species examined earlier [22,23] and confirm that birds demonstrate higher recombination rates than mammals and other vertebrates [3]. This can be partially explained by the high diploid chromosome number (most bird species have 2n = 78 ± 2) and the assurance of at least one crossing-over per bivalent for correct segregation. However, the number of chromosome arms could probably serve as a better rough estimate of the lower limit of crossover number per cell in birds. Indeed, *bullfinch*, *goldfinch*, and *linnet*, as well as several bird species studied earlier, exhibit a level of recombination comparable to the haploid arm number of 50 ± 3 [23,46,49]. This suggestion is also supported by the absence of transcentromere interference in bivalent 1 observed in our study. We detected no correlation between the positions of the neighboring MLH1 foci across the centromere (Table 3). Bivalents 1 lacking MLH1 foci at one of the arms were extremely rare. We suggest that the arm-specific assurance in the examined bivalent is due to the recombination independence of the arms, such that, at least in a given bivalent, recombination events do not interfere across the centromere.

At the same time, many phylogenetically unrelated bird species (including *siskin* from our study) demonstrate a recombination rate significantly higher than the haploid arm number: up to 65 in male chickens [50] or 76.1 in female white wagtails [22]. This diversity underscores the substantial variation in recombination rate among birds and suggests a potential role for selection in driving this variation. Despite a relatively recent divergence and karyotypic similarity, the cardueline species examined here showed significant differences in the recombination rate (Table 1). These differences do not demonstrate a concordance with the phylogenetic distances between the species. Closely related *siskin* and *goldfinch* differ more than phylogenetically more distant *linnet* and *bullfinch* (Figure 1, Table 1). The adaptive significance of the differences is obscure. The *siskin* has the highest recombination rate and the smallest area of distribution, while the *bullfinch* with the lowest recombination rate occupies the largest area among the four species examined [51]. However, the small number of species analyzed does not allow us to reveal the cause of the differences. Future comparative analyses of recombination rates and their interplay with environmental factors and life-history traits across diverse avian species will shed light on the mechanisms and causes of variation in recombination rates in birds.

The interspecies differences in the number of recombination events on bivalent 1 were correlated with the total recombination rates (Table 1 and Table 2). The recombination landscape of bivalent 1 was rather similar in the species examined and resembled that described previously in most vertebrates: telomeric peaks and valleys near the centromeres [17,21,52,53]. Let us consider how the centromere effect and interference contribute to the crossover distribution observed in our study.

The increased frequency of recombination near telomeres is usually explained by the formation of a telomeric bouquet in early leptotene. This conservative structure facilitates homology search and the formation of recombination intermediates [54]. However, the control over their resolution via crossover or noncrossover pathways apparently differs between the species and depends on the number of crossovers. Thus, the single foci at each arm in all SC1 examined here were located near the telomeric ends of the bivalents (Figure 7) similar to the recombination pattern observed in two anole lizards [55]. However, this is not the case in many other species examined. For example, in *Bovides* [56], *mice* [57], and *shrews* [45] single recombination events are usually located in the middle of the chromosome arms, both in metacentric and acrocentric chromosomes.

The distribution of multiple crossovers per arm or bivalent is usually explained by the effect of interference preventing neighbor crossovers from occurring close to each other. In most species, it results in the formation of two or more recombination peaks shifted towards the telomere and centromere. The centromere peak is usually lower than the telomere one, and recombination is often suppressed in the close vicinity of the centromere.

The suppression of recombination in the centromeric region was first described in the 1930s and referred to as the “centromere effect” [58,59]. It was believed to be caused by the condensed state of centromeric heterochromatin compared to the euchromatic regions of chromosomes [60,61,62]. However, later evidence suggested the existence of other mechanisms responsible for recombination suppression in the centromeric region [16], including a reduced number of recombination hotspots in the pericentromeric chromatin [63,64] or/and their accessibility due to epigenetic modification of the pericentromeric heterochromatin [65,66].

In the species examined here, a greater number of crossovers per arm leads to proximal crossovers occurring closer to the centromere. Additional recombination peaks appeared noticeably lower than telomeric ones. Centromeric and pericentromeric regions of pachytene bivalents (with the possible exception of the ZZ bivalent) were heterochromatic, as evidenced by the labeling of H3K9me3 (Figure 5). However, we did not detect transcentromere interference for the crossovers located on different arms of SC1.

We suggest that this might be explained via the noticeable shift toward the telomere’s distribution of recombination events. Apparently, in the birds examined, the initiation of recombination is systematically biased toward the subtelomeric regions, and interference pushes the subsequent recombination sites from the telomeres up to the centromeres. Thus, the deficiency of recombination sites around the centromere does not demand a special suppressive effect exerted by the centromere itself, although it does not exclude it.

Although the bird metapolycentromeres do not seem to affect the recombination landscape, they are an interesting (and rare) cytogenetic phenomenon deserving special attention. Centromeric protein-associated domains in avian metapolycentromeres are distributed in a paired bead-like pattern (Figure 2A,B and Figure 4) characteristic of all described species with metapolycentromeres [29,30,40,41]. The fusion of centromeric domains (Figure 4B) and the formation of a filament-like pattern have also been observed in some legume metapolycentromeres [29]. However, wide gaps between the domains (Figure 4A), the unequal number of domains, and their offset relative to each other in lateral elements of the synapsed homologous chromosomes (Figure 4C) have never been observed before and pose interesting questions. Do the gaps between the domains reflect the presence of sequences unable to form kinetochores, or are they just the artifacts of the SC stretching? Is the difference between the homologs in the domain number genetic or epigenetic? Molecular genetic and cytological analysis of mitotic chromosomes might help to answer these questions and shed light on the origin of metapolycentromeres in songbirds.

## 5. Conclusions

For the first time, we estimated the average recombination rate in the males of four species of finches. In spite of their relatively recent divergence and karyotypic similarity, they showed significant differences in the recombination rate. These data, together with earlier published MLH1-based estimates of male recombination rates of several other species of different bird orders, might provide a background for future studies into the evolution of the recombination rate in birds.

At the macrochromosome 1 of all species examined, we observed a recombination pattern similar to that described in many vertebrate species. More recombination nodules were located at the chromosome ends, with fewer near the centromeres. The distance of the proximal recombination nodules to the centromere is probably determined by the number of nodules that occurred on the chromosome arm; the more nodules, the closer they are. This pattern is likely due to the necessity of having at least one recombination event on each chromosome arm and interference between these events to prevent them from occurring too close together. The macrochromosomes 1 of two species had metapolycentromeres made up of multiple centromeric domains, while those of two other species had regional centromeres. However, the type of centromere (metapolycentromere or regional) did not influence the recombination pattern of the chromosome.

We propose that the limited occurrence of recombination sites near centromeres may result from the sequential process of crossover initiation from the chromosome ends rather than from the suppressive influence exerted by the centromere itself.

## Figures and Tables

**Table 1:** Recombination-related traits in four species of finches.

Species	N Birds	N Cells	MLH1 Focus Number per Cell	Total SC Length (µm)
*Linnet*	3	114	52.9 ± 2.8	313.3 ± 60.1
*Bullfinch*	4	177	49.5 ± 3.5	219.8 ± 41.3
*Siskin*	3	163	61.5 ± 6.3	245.1 ± 50.9
*Goldfinch*	3	119	52.2 ± 2.7	209.5 ± 28.8

**Table 2:** Recombination-related traits of arms of the SC1 in four species of finches.

Species	N Cells	Relative Length of the SC1 *	Centromeric Index	SC Arm	Mean MLH1 Focus Number	SC Length, μm	Mean Relative Distance ** between Neighboring MLH1 Foci
*Linnet*	114	0.11 ± 0.01	0.33 ± 0.04	*p*	1.2 ± 0.4	10.7 ± 2.8	0.57 ± 0.02
				*q*	2.2 ± 0.7	22.9 ± 6.0	0.43 ± 0.03
*Bullfinch*	164	0.11 ± 0.01	0.37 ± 0.03	*p*	1.2 ± 0.4	8.1 ± 1.9	0.51 ± 0.02
				*q*	1.7 ± 0.6	14.3 ± 2.8	0.50 ± 0.03
*Siskin*	157	0.11 ± 0.01	0.34 ± 0.03	*p*	1.9 ± 0.6	9.1 ± 2.3	0.45 ± 0.02
				*q*	2.9 ± 0.9	18.1 ± 4.8	0.31 ± 0.02
*Goldfinch*	102	0.11 ± 0.01	0.32 ± 0.03	*p*	1.1 ± 0.3	7.4 ± 1.5	0.58 ± 0.02
				*q*	1.4 ± 0.6	15.8 ± 3.6	0.39 ± 0.03

* fraction of the total SC length per cell. ** fraction of the arm length (excluding the centromere region in species with metapolycentromeres).

**Table 3:** Coefficients of correlation (R) between the distances from the centromere to the neighboring MLH1 foci within the arm and across the centromere.

Species	R within the Arm	R across the Centromere
*Linnet*	0.60 *	0.03
*Bullfinch*	0.30 *	0.09
*Siskin*	0.70 *	−0.01
*Goldfinch*	0.25	0.00

* Correlation coefficients significantly different from zero (*p* < 0.01).

## Data Availability

The data presented in this study are available in the article and Supplementary Materials.

## Supplementary Materials

The following supporting information can be downloaded at: https://www.mdpi.com/article/10.3390/ani13233624/s1. All numerical data used in this paper are present in the supplementary Excel file Supplimentary_tables.xlsx.
